# Editorial: Emerging Viruses: Host Immunity and Novel Therapeutic Interventions

**DOI:** 10.3389/fimmu.2018.02828

**Published:** 2018-11-30

**Authors:** Anna Smed-Sörensen, Ding Yuan Oh, Hiroyuki Oshiumi, Alan Chen-Yu Hsu

**Affiliations:** ^1^Division of Immunology and Allergy, Department of Medicine Solna, Karolinska Institutet, Karolinska University Hospital, Stockholm, Sweden; ^2^School of Health and Life Sciences, Federation University, Gippsland, VIC, Australia; ^3^Department of Immunology, Faculty of Life Sciences, Kumamoto University, Kumamoto, Japan; ^4^Viruses, Infections/Immunity, Vaccines & Asthma, Hunter Medical Research Institute, Newcastle, NSW, Australia; ^5^Faculty of Health & Medicine, Priority Research Centre for Healthy Lungs, The University of Newcastle, Newcastle, NSW, Australia

**Keywords:** influenza virus, coronavirus, MERS coronavirus, Ebola, Flavivirus, Hantavirus, vaccines, antiviral drugs

The inevitable emergence of novel infectious viruses and their unpredictable pathogenicity, transmissibility, and pandemic potentials are a major threat to human health. In particular, highly pathogenic influenza A viruses (IAVs), severe acute respiratory syndrome- and Middle East respiratory syndrome-coronavirus (SARS-CoV; MERS-CoV), Ebola virus (EBOV), and mosquito-borne Zika virus (ZIKV; Flavivirus) have attracted the world's attention due to their high pathogenicity, transmission and high mortality. While these viruses are mostly found in animals they can cause diseases and death in humans (zoonosis) when transmitted via close contact. Transmission of these pathogens is likely worsened by globalization and close quarter living in densely populated areas, all of which promotes rapid virus evolution and pandemic potential in humans. Significant research has resulted in deeper understanding of the virology, virus-host interactions, and disease pathology. These investigations have led to the development of vaccines and antiviral drugs, particularly for IAVs, and experimental vaccines for EBOV. Nevertheless, rapid and frequent virus mutations/reassortment often render current therapeutics ineffective, highlighting the limitations of our current therapeutic design strategies. Development of novel prevention and treatment options against these continuously mutating viruses must be explored before the next pandemic occurs.

In this Research Topic, a series of articles provides comprehensive insights on the current view of the virology, innate immune responses, and novel therapeutics to IAVs, MERS-CoV, EBOV, and flavivirus in experimental settings as well as in clinical trials.

In original research articles, Westenius et al. demonstrate that the highly pathogenic avian IAV H5N1 but not H3N2 and H7N9 virus replicate efficiently in primary human dendritic cells (DCs) and macrophages despite the robust induction of antiviral cytokines. This indicates an unusual level of resistance to host antiviral responses by the IAV H5N1 subtype. Prescott et al. show that MERS-CoV infection leads to much higher viral replication in the immuno-compromised rhesus macaque model, although this is accompanied by milder pathology in airways compared with non-immunocompromised control animals. This indicates that MERS-CoV infection in healthy individuals causes severe pathological changes with increased inflammatory response and cellular infiltrates in the airways. This is consistent with the finding that MERS-CoV-infected human patients have increased numbers of neutrophils and macrophages in their bronchial lavage fluid (Prescott et al.). Strandin et al. also show that the levels of neutrophils and the pro-inflammatory cytokine IL-8 are substantially higher in the blood of patients with hantavirus infection-mediated haemorrhagic fever with renal syndrome (HFRS). Neutrophil extracellular trap (NET) activation is evident and is the result of hantavirus-infected microvascular endothelial cells, indicating the importance of neutrophils in the disease pathology driven by both MERS-CoV and hantavirus infections.

This Research Topic also features a number of Review Articles on IAVs, flaviviruses, and EBOV, innate immune responses to infections, and novel therapeutic strategies that are currently in experimental phase or human clinical trials. Dou et al. provide a detailed review on IAV entry, viral replication, viral assembly and budding process, while Horman et al. review the IAV fitness, clinical manifestation of the disease, pathogenesis of highly pathogenic IAV infections. Hsu reviews the essential mutations and IAV virulence factors that are important in IAV transmission, inflammatory cytokine storm and efficient suppression of host antiviral response. Immune cells, such as macrophages and DCs are important in the immediate control of viral replication in the airways and also in establishing appropriate adaptive immune response for efficient clearance of the virus. Vangeti et al. discuss how these immune cells contribute to increased inflammation and severe disease caused by IAV. The prolonged inflammation and severe pathologies in the airways are not only observed with IAV, but also with MERS-CoV and hantavirus infections. Micro-RNAs (miRNAs; miRs) are a novel class of immuno-regulators that have been shown to be involved in innate immune responses. Nguyen et al. review several miRNAs that are highly induced by IAVs and directly promotes nuclear-factor-kappa-B (NF-κB)-mediated inflammatory response.

Flavivirus, such as Dengue virus (DENV) and recently emerged ZIKV are mosquito-borne infectious pathogens. Laureti et al. critically review important Flavivirus species, including DENV, Japanese encephalitis virus (JEV), ZIKV, and yellow fever virus (YFV), their binding receptor diversity and virus entry mechanisms. Blom et al. review the innate immune responses and immuno-pathologies induced by these pathogens.

In terms of therapeutics, virus-targeted strategies remain a popular approach. Valkenburg et al. review the criteria, strategies, and obstacles in the development of universal influenza vaccines, and the requirement to increase the strength and duration of vaccine-induced immune responses. In addition to vaccines, Davidson reviews the potentials of several IAV haemagglutinin (HA)-targeted monoclonal antibodies and viral polymerase inhibitory compounds that show promising therapeutic effects in pre-clinical or clinical trials. Lee et al. review DNA vaccines carrying various chimeric fusion proteins of conserved regions of IAV structural proteins and their effectiveness in inducing cross-reactive antibody response to different subtypes of IAVs. While universal IAV vaccines are still in experimental/clinical phase, vaccines targeting one flavivirus species have been shown to induce cross-reactive response against another viral species. Blom et al. review T cell cross-reactivity induced by JEV- and YFV-vaccine to DENV and ZIKA, respectively, indicating the potential use of JEV or YFV vaccine as protective therapeutics against ZIKA.

Viruses, such as IAVs undergo rapid virus mutations that often render vaccines and antiviral drugs less effective. Alternative host-targeted approaches are also extensively reviewed in this Research Topic as strategies to improve anti-viral therapy. HA cleavage and activation by host proteases is a critical step to rendering newly made IAV particles infectious. Yip et al. review a number of clinically used protease inhibitors currently used for diseases, such as liver fibrosis and cancer that also inhibit HA activation in *in vitro* and/or *in vivo* models. This highlights the potential of repurposing these compounds as antivirals drugs against IAVs. Hsu also reviews a number of peptide-based small molecules that inhibit HA-mediated viral internalization and reduce infection, and many of which are currently in the experimental phase of testing and in human clinical trials.

As disease pathologies are mostly driven by exaggerated immune responses, immuno-modulatory molecules have also been discussed as potential therapeutics to reduce tissue damage. Nguyen et al. review various miRNA inhibitors shown to directly suppress IAV replication, as well as those that reduce IAV-induced inflammatory cytokine storm in *in vivo* models. As an exaggerated inflammatory response appears to be a common phenomenon driven by most of the infectious viruses described here, they may also be applicable to other viral infections, such as MERS-CoV as treatments. Antiviral responses are critical in the immediate control of viral replication and are induced by the binding of host pattern recognition receptors to viral RNAs. Viruses, such as IAVs and EBOV produce virulence factors that inhibit the production of antiviral cytokines (Hsu, Dhama). Yong et al. review the use of synthetic virus RNA analogs and small molecule modulators as pan-antiviral drugs and vaccine adjuvants that boost antiviral responses against RNA viruses, such as IAVs or flavivirus.

The recent Ebola epidemics in Africa during 2014–2016 and in 2018 have raised serious concerns of EBOV infection as a global health threat due to its high mortality rate. Dhama et al. not only review the general virology of EBOV and disease progression, but also discuss current progress in the development of virus-, DNA-, and plant-based vaccines and treatment-based therapeutics that are urgently needed to prevent or reduce EBOV-mediated disease and mortality.

Collectively, this Research Topic highlights the ease in which viruses are able to cause severe disease, and the complexities of virus-host interactions that impact both disease pathology and outcome. The knowledge acquired from the articles contained within this special issue may lead to the development of more specific peptide-based antiviral agents, monoclonal antibodies and novel vaccines that protect against infections in the future. Synthetic- and host-RNA-based immuno-modulatory compounds may act as potential treatment that reduce symptoms and disease. As these are host-targeted they may be suited for multiple viral-induced diseases. This area of research is absolutely essential and is urgently required in preparation of future pandemics.

We wish to convey our appreciation to all the authors who have participated in this Research Topic and the reviewers for their insightful comments.

## Author contributions

All authors listed have made a substantial, direct and intellectual contribution to the work, and approved it for publication.

### Conflict of Interest Statement

The authors declare that the research was conducted in the absence of any commercial or financial relationships that could be construed as a potential conflict of interest.

